# Breastfeeding counseling in rooming-in care: a scoping review

**DOI:** 10.1590/0034-7167-2024-0187

**Published:** 2025-03-10

**Authors:** Michele Curcino Cavalcanti, Elisa da Conceição Rodrigues, Cynthya Viana de Resende, Monika Wernet, Karine Emanuelle Peixoto Oliveira da Silva, Mariana Torreglosa Ruiz

**Affiliations:** I Universidade Federal do Rio de Janeiro. Rio de Janeiro, Rio de Janeiro, Brazil; II Universidade Federal do Triângulo Mineiro. Uberaba, Minas Gerais, Brazil; III Universidade Federal de São Carlos. São Carlos, São Paulo, Brazil; IV Universidade Estadual de Feira de Santana. Feira de Santana, Bahia, Brazil

**Keywords:** Counseling, Breast Feeding, Rooming-in Care, Nursing Care, Review, Consejo, Lactancia Materna, Alojamiento Conjunto, Atención de Enfermería, Revisión

## Abstract

**Objectives:**

to map evidence available in the literature on breastfeeding counseling in rooming-in.

**Methods:**

a scoping review, following the stages recommended by the JBI to answer the review question: what evidence is available in the literature on breastfeeding counseling during the binomial’s hospitalization in rooming-in? Searches were conducted in the PubMed/MEDLINE, LILACS, Scopus, EMBASE, Web of Science, CINAHL, PsycInfo and CAPES Dissertation and Theses Catalogue databases, using the descriptors “Counseling”, “Breast Feeding”, “Rooming-in Care” and “Mother-Child Binomial” and their synonyms, without time or language limitations.

**Results:**

seven studies, published between 1999 and 2020, predominantly in English, were included in the analysis.

**Conclusions:**

there was a shortage of studies on the subject and the positive effects on reducing the belief of insufficient milk and singularities.

## RESUMEN

**Objetivos:**

mapear la evidencia disponible en la literatura sobre el asesoramiento sobre lactancia materna en alojamiento conjunto.

**Métodos:**

revisión del alcance, siguiendo los pasos recomendados por el JBI para responder a la pregunta de revisión: ¿qué evidencia está disponible en la literatura sobre el asesoramiento sobre lactancia materna durante la hospitalización del binomio en alojamiento conjunto? Las búsquedas se realizaron en las bases de datos PubMed/MEDLINE, LILACS, Scopus, EMBASE, *Web of Science*, CINAHL, PsycInfo y el Catálogo de Tesis y Disertaciones CAPES. utilizando los descriptores “*Counseling*”, “*Breast Feeding*”, “*Rooming-in Care*” e “*Mother-Child Binomial*” y sus sinónimos, sin delimitación de tiempo e idioma.

**Resultados:**

se incluyeron en el análisis siete estudios, publicados entre 1999 y 2020, predominantemente en inglés.

**Conclusiones:**

faltaron estudios sobre el tema y los efectos positivos en la reducción de la creencia de leche insuficiente y singularidades.

## INTRODUCTION

Breastfeeding (BF) promotes mother-baby bonding, reduces infant mortality and illness, increases intelligence levels, and reduces ovarian and breast cancer rates^(1)^. Human milk is the appropriate food for newborns (NBs) and children, fully meeting their nutritional needs up to the sixth month of life, and has unique immune components^(2)^.

The World Health Organization (WHO), the Ministry of Health (MoH) and the United Nations Children’s Fund (UNICEF) recognize BF as a promoter and protector of child development, recommended exclusively until the child’s sixth month of life and, in mixed form (concomitant with the introduction of solid foods), until two years of age or more^(3)^. However, BF is not biologically determined, since several psychosocial factors directly influence women’s decision, giving an extremely complex character to the BF process.

Rooming-in (RI) is the place where the binomial, woman and NB, both clinically stable, remain together from birth to hospital discharge, and is considered a strategic place and time for their comprehensive care^(4)^. Standard care in these units consists of empowering women and providing support so that they can care for themselves and the child at home^(5)^. Among the duties of the care team, the promotion of BF on demand stands out, focusing on particular needs and individualized support to postpartum women and companions, through qualified listening and reception^(4)^.

The period of hospitalization of the binomial is crucial for BF, since BF is not an instinctive act. It is a complex, multifactorial and multilevel practice, and the time of initiation predicts its exclusivity and continuation^(6)^. Furthermore, approximately 50% of women have great difficulty BF in the first three days after birth^(7)^, indicating the need for support and assistance during this period.

Counseling is an advanced technique of interaction and communication, based on Carl Rogers’s needs-centered therapy (counseling) and non-judgmental listening. Its pillars are the skills of listening and understanding and of increasing trust and providing support^(8,9)^.

The WHO defines BF counseling as support for women and NBs provided by healthcare professionals, assisting women and their families in making decisions to overcome possible difficulties. It was first described in 1993. It is a process based on dialogic interaction between healthcare professionals (counselors) and women who breastfeed or intend to breastfeed. Thus, it aims to empower women to breastfeed, respecting their reality and personal desires^(8,9)^. Therefore, it consists of a horizontal and person-centered approach that goes beyond clinical management, health education and guidelines for successful BF^(8,9,10)^.

To implement BF counseling, healthcare professionals need specific training, with theoretical and practical hours ranging from 20 to 40 hours, with counseling skills being worked on. Although counseling is considered a light technology to support BF, not all support is provided through this approach^(8,9,10)^.

The most important aspect of counseling is to listen attentively to postpartum women and her support network, seeking to understand how they feel. To do this, the skills of listening, understanding, developing trust and providing support are used. In the skills of listening and understanding, one should: use useful nonverbal communication; always ask open-ended questions to encourage dialogue; use responses and gestures that demonstrate interest in speakers’ speech; return what postpartum women say with their own words; show empathy and avoid words that sound like judgment. To develop trust and provide support, one should: accept what women say and feel; recognize and praise what is being done correctly in relation to BF, focusing on positive points; offer practical help; offer a small amount of relevant information that meets the demand of the moment; use simple language; and offer one or two suggestions for improvement, as long as they do not sound like orders^(9,11)^.

Counseling is recognized as an effective public health intervention, with evidence of increased rates of any BF, as well as exclusive BF, as indicated by a systematic review study with meta-analysis^(10)^. However, despite the evidence, there is still scarce evidence on its effectiveness and its implementation in assisting the binomial during hospitalization in RI, even though it is considered a critical and crucial period for successful BF.

The relevance of this study consists in preparing a synthesis of knowledge about the application of BF counseling during hospitalization in RI, considered a strategic period of care for the binomial.

## OBJECTIVES

To map evidence available in the literature on BF counseling during the binomial’s hospitalization in RI.

## METHODS

### Ethical aspects

Since this was a study that used public domain data and did not involve human beings, there was no need for assessment by a Research Ethics Committee. However, it is worth noting that the studies selected for the final sample were duly referenced.

### Design

This is a scoping review, developed based on the JBI recommendations. Thus, the following stages were followed: (1) establishing the title and the review question based on the PCC mnemonic, where: P: Population, C: Concept and C: Context; (2) exploring the state of the art of the research problem by writing the review introduction; (3) defining the inclusion criteria; (4) outlining the search strategy (sources, descriptors and manual references from reading the selected studies); (5) selecting the source of evidence (examiner and protocol); (6) selecting the articles - a process guided by the Preferred Reporting Items for Systematic Reviews and Meta-Analysis (PRISMA-ScR) flowchart^(12)^; (7) data extraction; (8) evidence analysis; and (9) presentation of results in tabular form and through descriptive mapping. The review protocol was registered with the Open Science Framework (https://osf.io/q4786).

### Study period and place

To elaborate the review question, the mnemonic PCC was used, in which Population (P) was the binomials, Concept (C) was BF counseling and Context (C) was hospitalization in RI. Thus, the review question was: what evidence is available in the literature on BF counseling during the binomial’s hospitalization in RI?

The searches were conducted in November 2022 and updated in June 2024, independently, by two reviewers, one master’s student and one doctoral student. One reviewer has experience with search strategy and a training course for scoping reviews, and both are specialists in maternal and child health. The search was validated by a librarian. Searches were conducted in the US National Library of Medicine National Institutes of Health (MEDLINE/PubMed), Web of Science (WoS), Excerpta Medica dataBASE (EMBASE), SciVerse Scopus, Cumulative Index to Nursing and Allied Health Literature (CINAHL), and Latin American and Caribbean Literature in Health Sciences (LILACS), PsycInfo, and the *Coordenação de Aperfeiçoamento de Pessoal de Nível Superior* (CAPES, Coordination for the Improvement of Higher Education Personnel) Thesis and Dissertation Catalog databases, correlating the descriptors “Counseling,” “Breast Feeding,” “Lactation,” “Rooming-in-Care,” and “Mother-Child Binomial”. No date, language, and/or study design filters were applied. The process of developing the search strategies followed the Peer Review of Electronic Search Strategies (PRESS) recommendations, and are presented in Chart 1.

**Chart 1:** Database search strategies and numerical results obtained, Rio de Janeiro, Rio de Janeiro, Brasil, 2024

	SEARCH STRATEGIES	n
**PubMed**	(Counseling[mh] OR Counseling[tiab] OR Counselling[tiab] OR Support*[tiab] OR Recommendation*[tiab]) AND (Breast Feeding[mh] OR Breast Feeding[tiab] OR Breastfeeding[tiab] OR Breast Fed[tiab] OR "Breast Milk"[tiab] OR Milk Sharing[tiab] OR Suckling[tiab] OR Lactation[mj] OR Lactation[tiab] OR Wet Nursing[tiab] OR Mother-Child Binomial*[tiab]) AND (Rooming-in Care[mh] OR Rooming-in Care*[tiab] OR Rooming-in[tiab])	**228**
**EMBASE**	('counseling'/exp OR 'counseling':ti,ab OR 'counselling':ti,ab OR support*:ti,ab OR recommendation*:ti,ab) AND ('breast feeding'/exp OR 'breast feeding':ti,ab OR breastfeeding:ti,ab OR 'breast fed':ti,ab OR 'breast milk':ti,ab OR 'milk sharing':ti,ab OR 'lactation':ti,ab OR suckling:ti,ab OR "wet nursing":ti,ab OR "mother-child binomial*":ti,ab) AND ('newborn care'/mj OR 'rooming-in care*':ti,ab OR 'rooming-in':ti,ab) AND [embase]/lim NOT ([embase]/lim AND [medline]/lim)	**112**
**Scopus**	TITLE-ABS-KEY(Counseling OR Counselling OR Support* OR Recommendation*) AND TITLE-ABS- KEY("Breast Feeding" OR Breastfeeding OR "Breast Fed" OR "Breast Milk" OR "Milk Sharing" OR Suckling OR Lactation OR "Wet Nursing" OR "Mother-Child Binomial") AND TITLE-ABS- KEY("Rooming-in Care" OR "Rooming-in")	**249**
**WoS**	(ALL=(Counseling OR Counselling OR Support* OR Recommendation*)) AND (ALL=("Breast Feeding" OR Breastfeeding OR "Breast Fed" OR "Breast Milk" OR "Milk Sharing" OR Suckling OR Lactation OR "Wet Nursing" OR "Mother-Child Binomial")) AND (ALL=("Rooming-in Care" OR "Rooming-in"))	**193**
**CINAHL**	(Counseling OR Counselling OR Support* OR Recommendation*) AND ("Breast Feeding" OR Breastfeeding OR "Breast Fed" OR "Breast Milk" OR "Milk Sharing" OR Suckling OR Lactation OR "Wet Nursing" OR "Mother-Child Binomial") AND ("Rooming-in Care" OR "Rooming-in")	**143**
**LILACS**	(tw:(Counseling OR Counselling OR Support* OR Recommendation* OR Aconselhamento* OR Recomendac* OR Asesoramiento* OR Recomendacion*)) AND (tw:("Breast Feeding" OR Breastfeeding OR "Breast Fed" OR "Breast Milk" OR "Milk Sharing" OR Lactation OR Suckling OR "Wet Nursing" OR "Mother-Child Binomial" OR Aleitamento OR "Leite Materno" OR Lactação OR Lactante OR "Binomio mãe-filho" OR "Leche materna" OR Lactancia OR "Binomio madre-hijo")) AND (tw:("Rooming-in Care" OR "Rooming-in" OR "Alojamento Conjunto" OR "Alojamiento conjunto")) AND (db:("LILACS"))	**93**
**PsycInfo**	(Counseling OR Counselling OR Support* OR Recommendation*) AND ("Breast Feeding" OR Breastfeeding OR "Breast Fed" OR "Breast Milk" OR "Milk Sharing" OR Suckling OR Lactation OR "Wet Nursing" OR "Mother-Child Binomial") AND ("Rooming-in Care" OR "Rooming-in")	**31**
**CAPES**	Counseling AND Breast Feeding AND Rooming-in	**01**

### Inclusion and exclusion criteria

Studies on the application of BF counseling during hospitalization in RI, without time or language limitations, were included. Duplicate articles in the databases, studies with secondary data (reviews), study protocols, opinion articles, editorials, consensus(es), response letters, letters to the editor and articles that did not answer the review question were excluded.

**Figure 1:**
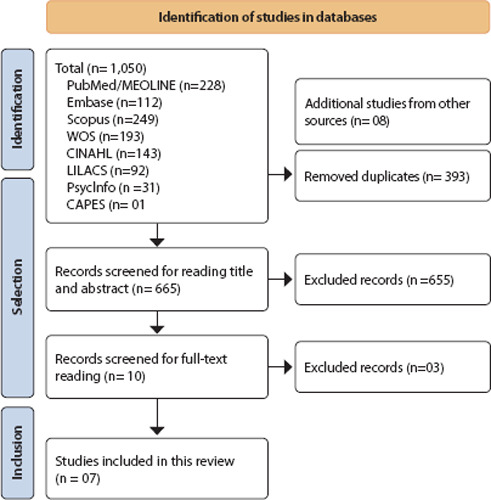
Flowchart according to Preferred Reporting Items for Systematic Reviews and Meta-Analysis (PRISMA-ScR) for study selection

The JBI Appraisal Tools^(13)^ methodological quality assessment tools were used to assess the methodological quality and risk of bias of included studies individually, using the versions specific to each type of study in order to qualify the included studies. This stage was performed by two researchers independently. It should be noted that this assessment was not considered a criterion for excluding the studies, but rather a qualifier of evidence, as this is a topic little explored in the literature. Figure 1 describes the result of the search with eligible primary studies and reasons for exclusion.

In the search, 1,058 studies were located. In the first stage, duplicates were removed (n = 393), and 655 articles were excluded after reading titles and abstracts because they did not portray the study topic. Afterwards, selected articles were read in full exhaustively. From the citation of the references of selected studies, it was possible to recover eight studies through the manual search. After exhaustive reading, the final sample consisted of seven studies.

### Study protocol

The studies were independently selected by two researchers, and disagreements were resolved by consensus. There was no need to include a third reviewer at this stage. The analysis of selected articles was performed, in a first stage, by reading title and abstract, followed by reading full text for the final selection of articles. The order of the databases analyzed was PubMed, EMBASE, Web of Science, CINAHL, LILACS, Scopus, PsycInfo and CAPES Thesis and Dissertation Catalog. Duplicate articles, study designs inappropriate for this review and those that did not answer the review question were excluded. Full texts were selected in a paired and independent manner, and those that met the eligibility criteria were selected for the study. Figure 1 illustrates the selection process of included studies.

### Analysis of results

Data were extracted as recommended by the JBI. When characterizing the studies, data on the author(s), year of publication, producing country, study objectives, population, sample size and methodology used were collected. When assessing the outcomes, details of the interventions performed and the results of the effectiveness of the application of BF counseling during the binomial’s hospitalization in RI were analyzed. The methodological quality was assessed using the JBI Appraisal Tools^(13)^, according to study design. A narrative data synthesis data from the articles was performed, which will be presented descriptively.

## RESULTS

Seven studies were included in the analysis, as shown in Chart 2. The first publication was dated 1999, and the last, from 2020, six (86%) published in English and one article (14%), provided by the authors, in Slovak. Canada, Denmark, Slovakia, United States, India, Iran and Sweden had one publication included each (14%).

**Chart 2:** Characteristics of studies included in the review (N = 07), 2024

Author(s)	Year of publication	Country	Objectives	Population and sample	Methodology	Intervention and comparator	Results	Risk of bias
Blixt I, Mårtensson LB & Ekström AC^(18)^	2014	Sweden	Assess the effect of counseling on women’s satisfaction, belief in insufficient breast milk and nipple pain, and duration of exclusive BF.	332 women 172 allocated to the intervention group (counseling) 160 to the control group – standard care	Longitudinal intervention study Healthy Swedish-speaking primiparous women who gave birth to healthy full-term babies were included, regardless of the mode of delivery.	Intervention: BF counseling in RI (trained nurses) Control: Standard care Three-day, three-month, and nine-month assessments	Counseling in RI influenced the satisfaction of postpartum women (p = 0.008), the feeling of coherence between guidelines and processes (p = 0.002) and the reduction in the belief of insufficient milk (p = 0.01) in the first three months.	6/8 (75%) – does not mention confounding factors and control measures for these factors.
Faridi, MMA & Dewan, P^(14)^	2008	India	Describe three cases of women who had nipple/breast malformations after counseling intervention.	03 postpartum women with nipple/breast malformations	Presentation of three cases Individual interventions using counseling skills, with the use of teaching materials when necessary. After discharge, follow-up visits were scheduled after one week and every three months.	Intervention: BF counseling in RI (trained nurses) Comparator: there were no comparators	A case of polymastia was reported, with maintenance of BF for four and a half months; a second case with flat nipples with an extensive burn scar that evolved with BF until the sixth month; and a third case with extensive burns on both breasts, with well-established BF on the fifth day and a confident mother.	7/8 (87.5%) - as there were no adverse events in the intervention, the item was not described in the case study.
Froonzani MD, Permehzadeh K, Motlagh ARD & Golestan B^(15)^	1999	Iran	Investigate the effect of BF education on exclusive BF rates, family nutrition and children’s health in the first four months of life.	120 postpartum women 59 allocated to the intervention group and 61 to the control group	Quasi-experimental study Public RI maternity wards Inclusion criteria were primiparous or never-breastfed women, healthy, who had delivered vaginally and given birth to healthy NBs. Post-discharge follow-up at ten, 15, 30 days and four months	Intervention: BF counseling in RI (trained nutritionist) Control: standard care	Higher rates of exclusive BF were observed in the study group (54%), compared to the control group (6.5%), at four months, fewer episodes of diarrhea in the study group (p = 0.004) and higher average weight and length (growth curve) in the babies in the study group.	9/9 (100%)
Gray KD e*t al*.^(17)^	2020	United States	Determine factors in hospital care that influence BF success.	7,370 binomials	Retrospective cohort study Inclusion criteria were mothers and healthy full-term NBs. Success was considered to be discharged on exclusive BF, without the use of formula.	Intervention: BF counseling in RI (consultants by International Board-Certified Lactation Consultants (IBCLCs) Comparator: standard care	Participants who received counseling were more likely to have successful BF (80%) than participants who received standard care (40%).	11/11 (100%)
Leonard LG(20)	2002	Canada	Describe a care plan for BF in cases of multiple pregnancies.	Not applicable	Descriptive theoretical study based on the author’s experience and scientific evidence	No interventions	The author reports positive results of BF counseling during hospitalization in RI in case of twin birth. In the end, he proposes a specific care plan.	6/6 (100%)
Nilsson et al.(16)	2017	Denmark	Assess the effect of BF counseling during hospitalization in RI on maternal BF self-efficacy, infant readmission and BF duration.	4,036 postpartum women 2,306 allocated to the intervention group and 1,730 to the control group	Randomized clinical trial in nine maternity hospitals Inclusion criteria were mothers of singletons, who intended to breastfeed, read and spoke Danish, and were not expected to be discharged after 50 hours postnatally due to pregnancy complications or clinical illness.	Intervention: BF counseling in RI (trained nurses) Comparator: standard care	Neonates in the intervention group had higher rates of exclusive BF at six months of age. Infants in the intervention group had lower hyperbilirubinemia scores and need for phototherapy treatment for jaundice.	9/12 (75%) - it was not possible to blind participants and researchers involved in the collection and study.
Schönbauerová A, Boledovičová M & Frčová B(19)	2020	Slovakia	Investigate maternal problems in BF, use and possibilities of counseling.	254 postpartum women	Cross-sectional study Inclusion criteria were mothers with children aged 12 to 24 months, aged over 18 years. Online data survey	No interventions	29.9% of mothers sought counseling services, 13.2% of them during their hospitalization in RI. More than half of the mothers reported problems due to the belief that BF was insufficient, and reported that the length of hospitalization and standard care were insufficient to maintain BF at home.	6/8 (75%) – does not mention confounding factors and control measures for these factors.

There was a diversity of designs, with one article being analyzed from each of the following modalities: longitudinal (14%); case series (14%); quasi-experimental (14%); descriptive (14%); cross-sectional (14%); and randomized clinical trial (14%).

Applying the JBI methodological quality and risk of bias assessment tools identified a low risk of bias (item compliance scores above 70%) in all included studies (100%). Three studies (42.8%) presented the description of all items to attest to the methodological quality according to the type.

From the analysis of outcomes: it was observed that counseling on BF in RI favored BF establishment and maintenance, including in cases of nipple/breast malformation or difficult management^(14)^; the chances of maintaining exclusive BF up to four^(15)^ and six months^(16)^ increased; the chances of using formulas during hospitalization in RI were reduced^(17)^; positive impacts were achieved on NBs’ health, such as fewer occurrences of diarrhea^(15)^, better outcomes in growth and development for age^(15)^; there were fewer occurrences of jaundice requiring phototherapy^(16)^. Thus, counseling on BF in RI proved to be an impactful strategy in reducing the belief that milk would be insufficient to nourish the NB^(17)^.

It is noteworthy that, among included studies, some described training the team for BF counseling; however, few described the counseling skills required in the interventions. Another highlight is the standard of care, since the studies do not detail the care routine used to assess and conduct BF in the RI space.

From study analysis, it was possible to extract two thematic categories, namely: 1. Counseling as a powerful strategy in managing the belief of insufficient milk; and 2. Counseling as welcoming singularities.

It was observed that the counseling provided during the binomial’s hospitalization in RI interfered with the deconstruction of the belief that it was insufficient to nourish a child with breast milk^(17, 18, 19)^, showing the importance of bets and investments for the development of nursing mothers’ skills and knowledge.

Although its applicability has no specificities, it has proven to be a powerful strategy in particular circumstances, such as in the case of twin pregnancies^(20)^, and in the approach to postpartum women with breast and/or nipple malformations and/or physiological or emotional difficulties in the BF process^(14)^. The review indicated the effectiveness of applying BF counseling during the binomial’s hospitalization in RI.

## DISCUSSION

Studies have shown the benefits of the counseling approach on BF during the binomial’s hospitalization in RI^(14, 15, 20)^.

Similarly, a study carried out in Iran with 68 postpartum women in RI, in which counseling was used, through communication skills, on maternal and neonatal care, through four individualized sessions lasting 60 to 90 minutes, with follow-up until the fourth week postpartum, having as a comparator the standard care offered in RI, where the topics of self-care, child care, including BF, mother-child interaction, support network, management and adaptation to motherhood were addressed, indicated that counseling contributed to empowerment and improved maternal performance^(21)^.

A review study, in which 5,084 binomials were assessed, indicated that BF counseling or consultancy by a trained professional increased early BF rates and improved all BF rates, including exclusive BF^(22)^. The lack of pre- and postnatal counseling was identified as an important barrier to BF^(23)^.

It is also worth noting that a quasi-experimental study with 234 women who received counseling through six consultations with an IBCLC consultant, one during pregnancy, in person in the first week after birth, telephone contacts at 14 and 28 days, in-person follow-up at five weeks and new telephone contact at ten weeks postpartum, showed an increase in the duration of BF and, mainly, its exclusive form^(24)^.

A study in which counseling was applied in four sessions with women who had previous unsuccessful experience of BF, with one in-person session during prenatal care, with groups of five to seven pregnant women, and telephone contacts at 15 days, two and four months postpartum, observed an increase in maternal self-efficacy and a greater ability to resolve breast problems and maintain BF^(25)^.

In this review, RI is considered strategic for the counseling approach. A study that assessed RI implementation in the United States^(26)^ indicated that, even with the adherence of most institutions, the binomial is still separated in the unit, whether for removing the baby for hearing tests, for heel punctures for glycemic control or venous punctures for sample collection, for baths, for pediatric physical examinations and even for taking photographs. The authors argue that efforts should be made to maintain the binomial in zero separation, since these moments can hinder the necessary support for BF and its establishment^(26)^.

A study with 1,080 women who intended to breastfeed during pregnancy highlighted the importance of support during hospitalization in RI for their training and empowerment to achieve their goals^(27)^. Similarly, a study conducted in Japan with more than 80 thousand binomials, which assessed the rate of exclusive BF in the sixth month, indicated that the factors that influenced maintenance were the earliest possible start of BF (first hour of life), skin-to-skin contact, social and financial support for postpartum women and length of stay in RI^(28)^. The authors also pointed out that smokers, obese women and those who needed to return to work were the ones who weaned their children early, signaling the need to look at these particularities while still in hospital^(28)^.

Hospital services directly influence BF rates, since this is the setting in which BF skills are first acquired. Postpartum women have a greater need for practical help; the importance of having BF assessed by a professional to establish BF; the relevance of guidance on the appropriate technique; and the impact of early initiation of BF (preferably within the first hour of life). Women who have undergone cesarean sections require greater practical help due to the post-surgical recovery process, and special attention should be given to nulliparous women, women who have undergone cesarean sections, and twin pregnancies. The study highlights the crucial role of RI in promoting and maintaining BF^(29)^.

This review revealed that counseling was a powerful strategy for managing the belief that there was insufficient milk. Worldwide^(30)^, the perception of insufficient milk is the main reason for stopping BF. It is common for mothers to associate NBs’ crying with low milk production^(30)^.

The counseling provided during prenatal care influenced the increase in maternal self-efficacy, a greater ability to overcome breast problems and the choice to continue BF in women with a history of difficulty in BF. In this study, in the assessment at 15 days postpartum, there was a greater description of nipple pain and trauma in the intervention group (counseling); however, at two and four months, approximately 93% of women in the intervention group were safe, the majority without breast problems and with sufficient breast milk production to meet NBs’ demand, while women in the control group accounted for 32% with the same characteristics^(25)^. Thus, counseling can influence postpartum woman in their empowerment, resilience and consequently, reduce the belief that their milk is insufficient, or if real insufficiency is detected, overcome this difficulty.

The review indicated that, although counseling is applicable to all binomials, it is particularly useful in specific cases, with emphasis on its use for women who have recently given birth to twins, with breast/nipple malformations and/or with physiological or emotional difficulties in the BF process. The literature also highlights the need for support through the counseling approach, especially for smokers^(28,31)^, obese women^(28,31)^, women who will return to work^(28)^, nulliparous women^(29,31)^, women who underwent cesarean section^(29)^, twin pregnancies^(29)^, with health problems and/or who need to use medications^(31)^, in cases of changes in the mechanics of BF by NBs (prematurity, fissures, ankyloglossia) and in cases of late lactogenesis^(31)^ and insufficient production^(31)^.

When analyzing a study, it was observed that the term “advice in BF” has been used, in some contexts, as a synonym for health education, guidance and clinical management^(32)^. This fact can lead to errors in mapping, interpreting and consolidating evidence on the topic and difficulties in incorporating counseling skills into institutional protocols and clinical practice.

A qualitative study with nurses who work in BF promotion showed that they are familiar with the strategies for clinical management of BF and that they approach it in a humanized manner. However, their actions are not systematized, and the focus is often restricted to guidance, with prioritization only for high-risk binomials^(33)^. Thus, the study points to the need for professional training for BF counseling, since it was identified that effective professional support is characterized by care provision from prenatal care by qualified and trained personnel, preferably in person, using counseling skills, with extension to the support network, and with a number of contacts between four and eight^(10)^.

### Study limitations

The scarcity of recent studies and publications on the subject as well as the greater detail of the protocols used in counseling compromise the comparability of the results found, which constitutes a limitation of this study, while at the same time pointing to the need and potential for new studies on the subject.

### Contributions to nursing, health or public policy

The review allowed mapping and exploring studies on counseling and its application during the binomial’s hospitalization in RI, which can guide its application in practice, due to the benefits of the strategy, as well as serving as a starting point for new research.

## CONCLUSIONS

There was a shortage of studies exploring the application of BF counseling in RI. Evidence indicated positive effects of counseling on BF indicators, and the strategy proved to be a powerful tool for maternal empowerment and management of the belief of insufficient milk as well as its relevance in singularities. However, the concept of counseling was often used in studies as a synonym for health guidelines/education, which may reduce the effective proposal of the concept of counseling. It is also worth highlighting the scarcity of descriptions of counseling protocols and interventions considered standard in hospital institutions and the need for new studies on the subject.
