# Supplementary material for: Breastfeeding counseling in rooming-in care: a scoping review
Source: Rev Bras Enferm. 2025 Mar 10;78(1):e20240187. doi: 10.1590/0034-7167-2024-0187 (PMC11893056; doi:10.1590/0034-7167-2024-0187)
Supplement: Translation

# Translation: Aconselhamento em aleitamento materno no alojamento conjunto: revisão de escopo

**DOI:** 10.1590/0034-7167-2024-0187pt

**Authors:** Michele Curcino Cavalcanti, Elisa da Conceição Rodrigues, Cynthya Viana de Resende, Monika Wernet, Karine Emanuelle Peixoto Oliveira da Silva, Mariana Torreglosa Ruiz

**Affiliations:** I Universidade Federal do Rio de Janeiro. Rio de Janeiro, Rio de Janeiro, Brasil; II Universidade Federal do Triângulo Mineiro. Uberaba, Minas Gerais, Brasil; III Universidade Federal de São Carlos. São Carlos, São Paulo, Brasil; IV Universidade Estadual de Feira de Santana. Feira de Santana, Bahia, Brasil

**Keywords:** Aconselhamento, Aleitamento Materno, Alojamento Conjunto, Cuidados de Enfermagem, Revisão

## Abstract

**Objetivos:**

mapear as evidências disponíveis na literatura sobre o aconselhamento em aleitamento materno no alojamento conjunto.

**Métodos:**

revisão de escopo, seguindo as etapas recomendadas pelo JBI para responder à questão de revisão: quais as evidências disponíveis na literatura sobre o aconselhamento em aleitamento materno durante a internação do binômio no alojamento conjunto? Buscas foram realizadas nas bases de dados PubMed/MEDLINE, LILACS, Scopus, EMBASE, *Web of Science*, CINAHL, PsycInfo e Catálogo de Teses e Dissertações da CAPES, utilizando os descritores “*Counseling*”, “*Breast Feeding*”, “*Rooming-in Care*” e *“Mother-Child Binomial”* e seus sinônimos, sem delimitação de tempo e idioma.

**Resultados:**

foram incluídos na análise sete estudos, publicados entre 1999 e 2020, com predomínio do idioma inglês.

**Conclusões:**

observaram-se escassez de estudos sobre a temática e os efeitos positivos na redução da crença de leite insuficiente e nas singularidades.

## INTRODUÇÃO

O aleitamento materno (AM) promove o vínculo mãe-bebê, reduz a mortalidade e o adoecimento infantil, aumenta os níveis de inteligência, além de reduzir as taxas de câncer de ovário e mama^(1)^. O leite humano é o alimento apropriado para o recém-nascido (RN) e criança, atendendo completamente as necessidades nutricionais até o sexto mês de vida, e possui componentes imunes exclusivos^(2)^.

A Organização Mundial da Saúde (OMS), o Ministério da Saúde (MS) e o Fundo das Nações Unidas para a Infância (UNICEF) reconhecem o AM como promotor e protetor do desenvolvimento infantil, recomendado na forma exclusiva até o sexto mês de vida da criança e, na forma mista (concomitante com a introdução alimentar), até os dois anos ou mais^(3)^. Contudo, o AM não é biologicamente determinado, uma vez que diversos fatores psicossociais influenciam diretamente a decisão da mulher, conferindo um caráter extremamente complexo ao processo de amamentar.

O alojamento conjunto (AC) é o local onde o binômio, mulher e RN, ambos clinicamente estáveis, permanecem juntos do nascimento à alta hospitalar, sendo considerado local e momento estratégico para sua assistência integral^(4)^. O cuidado padrão nessas unidades consiste em capacitar a mulher para emponderá-la e dar suporte para que ela possa cuidar de si e da criança no domicílio^(5)^. Entre as atribuições da equipe assistencial, destaca-se a promoção do AM sob livre demanda, com enfoque nas necessidades particulares e no apoio individualizado à puérpera e acompanhantes, através da escuta qualificada e acolhimento^(4)^.

O período de internação do binômio é crucial para a amamentação, uma vez que amamentar não é um ato instintivo. Trata-se de prática complexa, multifatorial e multinível, e o tempo de início prediz a sua exclusividade e continuação^(6)^. Além disso, cerca de 50% das mulheres têm muita dificuldade para amamentar nos primeiros três dias após o nascimento^(7)^, indicando necessidade de suporte e apoio nesse período.

O aconselhamento consiste em uma técnica avançada de interação e comunicação, tendo como base a terapia centrada nas necessidades do cliente (*counseling*) de Carl Rogers e na escuta sem julgamento. Tem como pilares as habilidades de escutar e compreender e de aumentar a confiança e dar apoio^(8,9)^.

A OMS define o aconselhamento em AM como suporte, para mulheres e RN, prestado por profissionais de saúde, auxiliando na tomada de decisão da mulher e sua família para superar as possíveis dificuldades, e sua primeira descrição foi datada de 1993. Trata-se de processo baseado na interação dialógica entre profissionais de saúde (conselheiros) e mulheres que amamentam ou pretendem amamentar. Assim, tem como objetivo capacitar a mulher para a amamentação, respeitando sua realidade e desejos pessoais^(8,9)^. Portanto, consiste em uma abordagem horizontal e centrada na pessoa que vai além do manejo clínico, da educação em saúde e das orientações para o sucesso do AM^(8,9,10)^.

Para implementar o aconselhamento em AM, os profissionais de saúde necessitam de formação específica, com carga horária teórica e prática variando de 20 a 40 horas, sendo trabalhadas as habilidades de aconselhamento. Embora o aconselhamento seja considerado uma tecnologia leve de suporte ao AM, nem todo suporte se dá através dessa abordagem^(8,9,10)^.

O aspecto primordial no aconselhamento é a escuta atenta à puérpera e sua rede de apoio, buscando compreender como se sentem. Para isso, são utilizadas as habilidades de escutar, compreender, desenvolver confiança e dar apoio. Nas habilidades de escutar e compreender, deve-se: usar comunicação não verbal útil; sempre fazer perguntas abertas para estimular o diálogo; utilizar respostas e gestos que demonstrem interesse pela fala do locutor; devolver com as palavras da própria puérpera o que ela diz; mostrar empatia e evitar palavras que soem como julgamento. Para desenvolver confiança e dar apoio, deve-se: aceitar o que a mulher diz e sente; reconhecer e elogiar o que está sendo feito corretamente em relação à amamentação, focando nos pontos positivos; oferecer ajuda prática; ofertar pouca e relevante informação que atenda à demanda do momento; usar linguagem simples; e oferecer uma ou duas sugestões para melhoria, desde que não soem como ordens^(9,11)^.

O aconselhamento é reconhecidamente uma intervenção eficaz em saúde pública, com evidências de aumento das taxas de qualquer aleitamento, bem como da amamentação exclusiva, conforme apontado por estudo de revisão sistemática com metanálise^(10)^. No entanto, apesar das evidências, ainda são escassas as evidências sobre sua eficácia e sua implementação na assistência à díade durante a internação no AC, mesmo sendo considerado um período crítico e crucial para o sucesso do aleitamento.

A relevância deste estudo consiste na elaboração da síntese do conhecimento sobre a aplicação do aconselhamento em AM durante a internação no AC, considerado período estratégico da assistência ao binômio.

## OBJETIVOS

Mapear as evidências disponíveis na literatura sobre o aconselhamento em AM durante a internação do binômio em AC.

## MÉTODOS

### Aspectos éticos

Por se tratar de estudo que utilizou dados de domínio público e não envolveu seres humanos, não houve necessidade de apreciação pelo Comitê de Ética em Pesquisa. Porém, cabe ressaltar que os estudos selecionados para a amostra final foram devidamente referenciados.

### Desenho

Trata-se de revisão de escopo, desenvolvida com base nas recomendações do JBI. Desse modo, percorreram-se as etapas de: (1) estabelecimento do título e da pergunta de revisão a partir do mnemônico PCC, sendo que: P: População, C: Conceito e C: Contexto; (2) exploração do estado da arte do problema de investigação com redação da introdução da revisão; (3) definição dos critérios de inclusão; (4) delineamento da estratégia de busca (fontes, descritores e referências manuais a partir da leitura das publicações selecionadas); (5) seleção da fonte de evidência (examinador e protocolo); (6) seleção dos artigos - processo guiado pelo fluxograma *Preferred Reporting Items for Systematic Reviews and Meta-Analysis* (PRISMA-ScR)^(12)^; (7) extração dos dados; (8) análise das evidências; e (9) apresentação dos resultados de forma tabular e por meio de mapeamento descritivo. O protocolo de revisão foi registrado na *Open Science Framework* (https://osf.io/q4786).

### Período e local do estudo

Para elaborar a questão de revisão, utilizou-se o mnemônico PCC, em que a População (P) foram os binômios, o Conceito (C) foi o aconselhamento em AM e o Contexto (C) foi a internação no AC. Dessa forma, a questão de revisão foi: quais as evidências disponíveis na literatura sobre o aconselhamento em AM durante a internação do binômio no AC?

As buscas foram realizadas em novembro de 2022 e atualizadas em junho de 2024, de forma independente, por dois revisores, um mestrando e um doutor. Um revisor tem experiência com estratégia de busca e curso de treinamento para *scoping reviews*, e ambos são especialistas na área da saúde materno-infantil. A busca foi validada por um bibliotecário. Foram realizadas buscas nas bases de dados *US National Library of Medicine National Institutes of Health* (MEDLINE/PubMed), *Web of Science* (WoS), *Excerpta Medica dataBASE* (EMBASE), SciVerse Scopus, *Cumulative Index to Nursing and Allied Health Literature* (CINAHL) e Literatura Latino-Americana e do Caribe em Ciências da Saúde (LILACS), PsycInfo e Catálogo de Teses e Dissertações da Coordenação de Aperfeiçoamento de Pessoal de Nível Superior (CAPES), correlacionando os descritores “*Counseling*”, “*Breast Feeding*”, “*Lactation*”, “*Rooming-in-Care*” e “*Mother-Child Binomial*”. Não foram aplicados filtros de data, idioma e/ou desenho de estudo. O processo de elaboração das estratégias de busca atendeu às recomendações do *Peer Review of Electronic Search Strategies* (PRESS), e são apresentadas no Quadro 1.

**Quadro 1:** Estratégias de buscas nas bases de dados e retorno numérico obtido, Rio de Janeiro, Rio de Janeiro, Brasil, 2024

	ESTRATÉGIAS DE BUSCA	n
**PubMed**	(Counseling[mh] OR Counseling[tiab] OR Counselling[tiab] OR Support*[tiab] OR Recommendation*[tiab]) AND (Breast Feeding[mh] OR Breast Feeding[tiab] OR Breastfeeding[tiab] OR Breast Fed[tiab] OR “Breast Milk”[tiab] OR Milk Sharing[tiab] OR Suckling[tiab] OR Lactation[mj] OR Lactation[tiab] OR Wet Nursing[tiab] OR Mother-Child Binomial*[tiab]) AND (Rooming-in Care[mh] OR Rooming-in Care*[tiab] OR Rooming-in[tiab])	**228**
**EMBASE**	(‘counseling’/exp OR ‘counseling’:ti,ab OR ‘counselling’:ti,ab OR support*:ti,ab OR recommendation*:ti,ab) AND (‘breast feeding’/exp OR ‘breast feeding’:ti,ab OR breastfeeding:ti,ab OR ‘breast fed’:ti,ab OR ‘breast milk’:ti,ab OR ‘milk sharing’:ti,ab OR ‘lactation’:ti,ab OR suckling:ti,ab OR “wet nursing”:ti,ab OR “mother-child binomial*”:ti,ab) AND (‘newborn care’/mj OR ‘rooming-in care*’:ti,ab OR ‘rooming-in’:ti,ab) AND [embase]/lim NOT ([embase]/lim AND [medline]/lim)	**112**
**Scopus**	TITLE-ABS-KEY(Counseling OR Counselling OR Support* OR Recommendation*) AND TITLE-ABS- KEY(“Breast Feeding” OR Breastfeeding OR “Breast Fed” OR “Breast Milk” OR “Milk Sharing” OR Suckling OR Lactation OR “Wet Nursing” OR “Mother-Child Binomial”) AND TITLE-ABS- KEY(“Rooming-in Care” OR “Rooming-in”)	**249**
**WoS**	(ALL=(Counseling OR Counselling OR Support* OR Recommendation*)) AND (ALL=(“Breast Feeding” OR Breastfeeding OR “Breast Fed” OR “Breast Milk” OR “Milk Sharing” OR Suckling OR Lactation OR “Wet Nursing” OR “Mother-Child Binomial”)) AND (ALL=(“Rooming-in Care” OR “Rooming-in”))	**193**
**CINAHL**	(Counseling OR Counselling OR Support* OR Recommendation*) AND (“Breast Feeding” OR Breastfeeding OR “Breast Fed” OR “Breast Milk” OR “Milk Sharing” OR Suckling OR Lactation OR “Wet Nursing” OR “Mother-Child Binomial”) AND (“Rooming-in Care” OR “Rooming-in”)	**143**
**LILACS**	(tw:(Counseling OR Counselling OR Support* OR Recommendation* OR Aconselhamento* OR Recomendac* OR Asesoramiento* OR Recomendacion*)) AND (tw:(“Breast Feeding” OR Breastfeeding OR “Breast Fed” OR “Breast Milk” OR “Milk Sharing” OR Lactation OR Suckling OR “Wet Nursing” OR “Mother-Child Binomial” OR Aleitamento *OR “*Leite Materno*” OR* Lactação *OR* Lactante *OR “*Binomio mãe-filho*” OR “Leche materna” OR Lactancia OR “Binomio madre-hijo”)) AND (tw:(“Rooming-in Care” OR “Rooming-in” OR “*Alojamento Conjunto*” OR “Alojamiento conjunto”)) AND (db:(“LILACS”))*	**93**
**PsycInfo**	(Counseling OR Counselling OR Support* OR Recommendation*) AND (“Breast Feeding” OR Breastfeeding OR “Breast Fed” OR “Breast Milk” OR “Milk Sharing” OR Suckling OR Lactation OR “Wet Nursing” OR “Mother-Child Binomial”) AND (“Rooming-in Care” OR “Rooming-in”)	**31**
**CAPES**	*Counseling AND Breast Feeding AND Rooming-in*	**01**

### Critérios de inclusão e de exclusão

Foram incluídos estudos sobre a aplicação do aconselhamento em AM durante a internação no AC, sem delimitação de tempo e idioma. Foram excluídos artigos duplicados nas bases, estudos com dados secundários (revisões), protocolos de estudos, artigos de opinião, editoriais, consenso(s), cartas resposta, cartas ao editor e artigos que não respondessem à questão de revisão.

As ferramentas de avaliação da qualidade metodológica do JBI *Appraisal Tools* ^(13)^ foram utilizadas para avaliar a qualidade metodológica e risco de viés dos estudos inclusos, individualmente, sendo utilizadas as versões próprias para cada tipo de estudo, a fim de qualificar os estudos incluídos. Esta etapa foi realizada por dois pesquisadores de forma independente. Ressalta-se que esta avaliação não foi considerada um critério de exclusão dos estudos, mas sim um qualificador da evidência, por se tratar de temática pouco explorada na literatura. O resultado da busca com os estudos primários elegíveis e motivos de exclusão é descrito na Figura 1.

**Figura 1:** Fluxograma segundo Preferred Reporting Items for Systematic Reviews and Meta-Analysis (PRISMA-ScR) para seleção dos estudos

Na busca, foram localizadas 1.058 publicações. Na primeira etapa, as duplicatas foram removidas (n = 393), e 655 artigos foram excluídos após a leitura dos títulos e resumos por não retratarem o tema de estudo. Após, os artigos selecionados foram lidos na íntegra exaustivamente. A partir da citação das referências dos estudos selecionados, foi possível recuperar oito estudos através da busca manual. Após a leitura exaustiva, a amostra final foi composta por sete estudos.

### Protocolo do estudo

A seleção dos estudos foi realizada, de modo independente, por dois pesquisadores, e as discordâncias foram resolvidas por consenso. Não houve necessidade de acréscimo do terceiro revisor nesta etapa. A análise dos artigos selecionados foi realizada, em uma primeira etapa, com a leitura do título e resumo, seguida de leitura na íntegra para a seleção final dos artigos. A ordem das bases de dados analisadas foi PubMed, EMBASE, *Web of Science*, CINAHL, LILACS, Scopus, PsycInfo e Catálogo de Teses e Dissertações da CAPES. Foram excluídos artigos duplicados, desenhos de estudo inadequados para esta revisão e não resposta à questão de revisão. Os textos completos foram selecionados de modo pareado e independente, e os que obedeceram aos critérios de elegibilidade foram selecionados para o estudo. A Figura 1 ilustra o processo de seleção dos estudos incluídos.

### Análise dos resultados

Os dados foram extraídos conforme recomendação do JBI. Na caracterização dos estudos, foram coletados os dados sobre autor(es), ano de publicação, país produtor, objetivos do estudo, população, tamanho amostral e metodologia utilizada. Na avaliação dos desfechos, foram analisados detalhamento das intervenções realizadas e os resultados da efetividade da aplicação do aconselhamento em AM durante a internação do binômio no AC. A qualidade metodológica foi avaliada por meio das ferramentas da JBI *Appraisal Tools* ^(13)^, de acordo com o tipo de estudo. Realizou-se a síntese narrativa dos dados dos artigos, os quais serão apresentados de forma descritiva.

## RESULTADOS

Foram incluídos na análise sete estudos, conforme apresentado no Quadro 2. A primeira publicação foi datada de 1999, e a última, de 2020, seis (86%) publicados no idioma inglês e um artigo (14%), cedido pelos autores, no idioma eslovaco. Canadá, Dinamarca, Eslováquia, Estados Unidos, Índia, Irã e Suécia tiveram uma publicação inclusa cada (14%).

**Quadro 2:** Características dos estudos inclusos na revisão (N = 07), 2024

Autor(es)	Ano de publicação	País	Objetivos	População e amostra	Metodologia	Intervenção e comparador	Resultados	Risco de viés
Blixt I, Mårtensson LB & Ekström AC(18)	2014	Suécia	Avaliar o efeito do aconselhamento na satisfação das mulheres, crença de leite materno insuficiente e dor nos mamilos e tempo de AM exclusivo.	332 mulheres 172 alocadas no grupo intervenção (aconselhamento) 160 no grupo controle – cuidado padrão	Estudo de intervenção longitudinal Incluíram-se primíparas saudáveis que falavam o idioma sueco e que deram à luz a bebês a termo e saudáveis, independentemente do tipo de parto.	Intervenção: aconselhamento em aleitamento no AC (enfermeiros capacitados) Controle: cuidado padrão Avaliações com três dias, três meses e nove meses	O aconselhamento no AC influenciou a satisfação das puérperas (p = 0,008), a sensação de coerência entre orientações e processos (p = 0,002) e a redução da crença de leite insuficiente (p= 0,01) nos primeiros três meses.	6/8 (75%) – não menciona fatores de confusão e medidas de controle para esses fatores.
Faridi, MMA & Dewan, P(14)	2008	Índia	Descrever três casos de mulheres que possuíam malformações mamilares/mamárias após intervenção de aconselhamento.	03 puérperas com malformações mamilares/mamárias	Apresentação de três casos Intervenções individuais usando as habilidades de aconselhamento, com uso de material didático quando necessário. Após a alta, foram agendados retornos com uma semana e a cada três meses para seguimento.	Intervenção: aconselhamento em aleitamento no AC (enfermeiros capacitados) Comparador: não houve comparadores	Relatado caso de polimastia, com manutenção do AM por quatro meses e meio; segundo caso com mamilos planos com cicatriz de queimadura extensa que evoluiu com AME até o sexto mês; e terceiro caso com queimaduras extensas em ambas as mamas, com amamentação bem estabelecida no quinto dia e mãe confiante.	7/8 (87,5%) - como não há eventos adversos na intervenção, o item não foi descrito no estudo de caso.
Froonzani MD, Permehzadeh K, Motlagh ARD & Golestan B(15)	1999	Irã	Investigar o efeito da educação em aleitamento nos índices de aleitamento exclusivo, na alimentação familiar e na saúde das crianças nos primeiros quatro meses de vida.	120 puérperas 59 alocadas no grupo intervenção e 61 no grupo controle	Estudo quase-experimental Enfermarias de AC de maternidade pública Critérios de inclusão foram primíparas ou mulheres que nunca tinham amamentado, saudáveis, que tiveram parto vaginal e deram à luz a neonatos saudáveis. Seguimento pós-alta com dez, 15, 30 dias e quatro meses	Intervenção: aconselhamento em aleitamento no AC (nutricionista capacitada) Controle: cuidado padrão	Observaram-se maiores taxas de aleitamento exclusivo no grupo de estudo (54%), comparado ao controle (6,5%), aos quatro meses, menos episódios de diarreia no grupo de estudo (p = 0,004) e maiores médias de peso e comprimento (curva de crescimento) nos bebês do grupo de estudo.	9/9 (100%)
Gray KD e*t al*.(17)	2020	Estados Unidos	Determinar fatores da assistência hospitalar que influenciam o sucesso da amamentação.	7.370 díades	Estudo de coorte retrospectiva Critérios de inclusão foram mães e RNs a termo e saudáveis. Considerou-se sucesso receber alta em AM exclusivo, sem uso de fórmula.	Intervenção: aconselhamento em aleitamento no AC (consultores pela *International Board-Certified Lactation Consultants* (IBCLCs) Comparador: cuidado padrão	Participantes submetidas ao aconselhamento tiveram maior probabilidade de ter amamentação bem-sucedida (80%) do que as participantes que receberam cuidado padrão (40%).	11/11 (100%)
Leonard LG(20)	2002	Canadá	Descrever um plano de cuidados para AM em casos de gestações múltiplas.	Não se aplica	Estudo teórico descritivo baseado na experiência do autor e nas evidências científicas	Sem intervenções	O autor reporta resultados positivos do aconselhamento em AM durante a internação no AC em caso de nascimento gemelar. Ao final, propõe um plano de cuidados específicos.	6/6 (100%)
Nilsson et al.(16)	2017	Dinamarca	Avaliar o efeito do aconselhamento em AM durante a internação no AC na autoeficácia materna em amamentar, reinternação infantil e duração da amamentação.	4.036 puérperas 2.306 alocadas no grupo intervenção e 1.730 no grupo controle	Ensaio clínico randomizado em nove maternidades Critérios de inclusão foram mães de neonatos únicos, que pretendiam amamentar, liam e falavam idioma dinamarquês, sem previsão de alta após 50 horas pós-natal devido a complicações na gravidez ou doença clínica.	Intervenção: aconselhamento em aleitamento no AC (enfermeiras capacitadas) Comparador: cuidado padrão	Neonatos do grupo intervenção apresentaram maiores taxas de AM exclusivo aos seis meses de vida. Lactentes do grupo intervenção tiveram menores escores de hiperbilirrubinemia e necessidade de tratamento com fototerapia para icterícia.	9/12 (75%) - não foi possível cegar participantes e pesquisadores envolvidos na coleta e estudo.
Schönbauerová A, Boledovičová M & Frčová B(19)	2020	Eslováquia	Investigar problemas maternos na amamentação, uso e possibilidades do aconselhamento.	254 puérperas	Estudo transversal Critérios de inclusão foram mães com filhos de 12 a 24 meses, com idade superior a 18 anos. Inquérito de dados *online*	Sem intervenções	29,9% das mães buscaram serviços de aconselhamento e 13,2% delas durante a internação no AC. Mais da metade das mães relatou problemas pela crença de amamentação insuficiente, e relatou que o tempo de internação e o cuidado padrão foram insuficientes para manutenção do AM no domícilio.	6/8 (75%) – não menciona fatores de confusão e medidas de controle para esses fatores.

Verificou-se diversidade de desenhos, sendo analisados um artigo de cada uma das modalidades: longitudinal (14%); série de casos (14%); quase-experimental (14%); descritivo (14%); transversal (14%); e ensaio clínico randômico (14%).

A aplicação das ferramentas de avaliação da qualidade metodológica e risco de viés do JBI identificou baixo risco de viés (pontuações de atendimento aos itens acima de 70%) em todos os estudos incluídos (100%). Três estudos (42,8%) apresentaram a descrição de todos os itens para atestar a qualidade metodológica conforme o tipo.

A partir da análise dos desfechos: observou-se que o aconselhamento em AM no AC favoreceu o estabelecimento e manutenção do AM, inclusive em casos de malformação mamilar/mamária ou de difícil manejo^(14)^; aumentaram-se as chances de manutenção do AM na forma exclusiva até os quatro^(15)^ e os seis meses^(16)^; reduziram-se as chances do uso de fórmulas durante a internação no AC^(17)^; alcançaram-se impactos positivos na saúde do neonato, tais como menores ocorrências de diarreia^(15)^, melhores desfechos em crescimento e desenvolvimento para a idade^(15)^; houve menores ocorrências de icterícia com necessidade de fototerapia^(16)^. Destarte, o aconselhamento em AM no AC mostrou-se uma estratégia de impacto na redução da crença de que o leite seria insuficiente para nutrir o neonato^(17)^.

Destaca-se que, entre os estudos inclusos, alguns descreveram a formação da equipe para o aconselhamento em AM, no entanto poucos descreveram as habilidades do aconselhamento requeridas nas intervenções. Outro ponto de destaque é o cuidado padrão, já que os estudos não detalham a rotina assistencial utilizada para avaliação e condução do AM no espaço do AC.

A partir da análise dos estudos, foi possível extrair duas categorias temáticas, a saber: 1. O aconselhamento como estratégia potente no manejo da crença de leite insuficiente; e 2. O aconselhamento enquanto acolhimento de singularidades.

Observou-se que o aconselhamento realizado durante a internação do binômio no AC interferiu na desconstrução da crença da insuficiência em nutrir a criança com o leite materno^(17,18,19)^, mostrando a importância de apostas e investimentos para o desenvolvimento de habilidades e conhecimentos da nutriz.

Embora sua aplicabilidade não tenha especificidades, mostrou-se uma estratégia potente nas particularidades, como no caso da gestação gemelar^(20)^, e na abordagem de puérperas com malformações nas mamas e/ou mamilares e/ou com dificuldades fisiológicas ou emocionais no processo de amamentação^(14)^. A revisão apontou a efetividade da aplicação do aconselhamento em AM durante a internação do binômio no AC.

## DISCUSSÃO

Estudos apontaram os benefícios da abordagem do aconselhamento sobre AM durante a internação do binômio no AC^(14,15,20)^.

Semelhantemente, estudo realizado no Irã com 68 puérperas no AC em que foi utilizado o aconselhamento, através das habilidades de comunicação, sobre cuidados maternos e neonatais, por meio de quatro sessões individualizadas com duração de 60 a 90 minutos, com seguimento até a quarta semana pós-parto, tendo como comparador o cuidado padrão ofertado no AC, onde foram abordados os temas sobre autocuidado, cuidado infantil, incluindo o AM, interação mãe-filho, rede de apoio, gestão e adaptação à maternidade, apontou que o aconselhamento contribuiu para o empoderamento e melhora do desempenho materno^(21)^.

Estudo de revisão, em que foram avaliados 5.084 binômios, apontou que o aconselhamento ou consultoria em aleitamento por profissional capacitado aumentou os índices de aleitamento precoce e melhorou todas as taxas de AM, incluindo o exclusivo^(22)^. Já a ausência de aconselhamento pré e pós-natal foi apontada como uma importante barreira para o AM^(23)^.

Ainda destaca-se que estudo quase-experimental, com 234 mulheres, que tiveram aconselhamento através de seis consultas com consultora do IBCLC, sendo uma durante a gestação, presencialmente na primeira semana pós-parto, contatos telefônicos com 14 e 28 dias, retorno presencial com cinco semanas e novo contato telefônico com dez semanas pós-parto, apontou aumento da duração do aleitamento e, principalmente, da sua forma exclusiva^(24)^.

Estudo em que foi aplicado o aconselhamento com quatro sessões com mulheres que tinham experiência prévia mal sucedida de AM, sendo uma sessão presencial durante o pré-natal, com grupos de cinco a sete gestantes, e contatos telefônicos aos 15 dias, dois e quatro meses pós-parto, observou aumento da autoeficácia materna e maior capacidade de resolver problemas mamários e manter o aleitamento^(25)^.

Na presente revisão, o AC é considerado estratégico para a abordagem do aconselhamento. Estudo que avaliou a implantação do AC nos Estados Unidos^(26)^ apontou que, mesmo diante da adesão da maioria das instituições, o binômio ainda é separado na unidade, quer seja para a remoção do bebê para testes auditivos, para punções calcâneas para controle glicêmico ou venosa para coleta de exames, para banhos, para realização de exame físico pediátrico e até mesmo para realização de fotografias. Os autores defendem que devem ser feitos esforços para manter o binômio em separação zero, uma vez que esses momentos podem prejudicar o suporte necessário ao AM e seu estabelecimento^(26)^.

Estudo com 1.080 mulheres, que tinham intenção de amamentar durante a gestação, apontou a importância do apoio durante a internação no AC para sua formação e empoderamento para atingir seus objetivos^(27)^. Semelhantemente, estudo realizado no Japão com mais de 80 mil binômios, que avaliou o índice de AM exclusivo no sexto mês, apontou que os fatores que influenciaram a manutenção foram o início mais precoce possível do AM (primeira hora de vida), o contato pele-a-pele, apoio social e financeiro da puérpera e o tempo de permanência no AC^(28)^. Os autores apontaram ainda que mulheres tabagistas, obesas e as que necessitaram voltar ao trabalho foram as que desmamaram precocemente seus filhos, sinalizando a necessidade do olhar para essas particularidades ainda na internação^(28)^.

Os serviços hospitalares influenciam diretamente os índices de AM, uma vez que, neste cenário, ocorre o início da aquisição de habilidades para o aleitamento. As puérperas apresentam maior necessidade de ajuda prática; a importância da avaliação das mamadas realizada por profissional para seu estabelecimento; a relevância da orientação da técnica adequada; e o impacto do início precoce do aleitamento (preferencialmente na primeira hora de vida). Mulheres submetidas à cesárea, devido ao processo de recuperação pós-cirúrgico demandam maior ajuda prática, e deve ser dada especial atenção nos casos de nulíparas, mulheres submetidas à cesárea e gestações gemelares. O estudo aponta para a crucialidade do AC para a promoção e manutenção do aleitamento^(29)^.

Identificou-se, através da presente revisão, que o aconselhamento foi uma estratégia potente no manejo da crença de leite insuficiente. Mundialmente^(30)^, a percepção de leite insuficiente é a principal razão para a interrupção do AM. É comum as mães associarem o choro do RN à baixa produção láctea^(30)^.

O aconselhamento fornecido no pré-natal influenciou o aumento da autoeficácia materna, maior capacidade de superar problemas mamários e optar por manter o aleitamento em mulheres com história de dificuldade em amamentar. Neste estudo, na avaliação aos 15 dias pós-parto, houve maior descrição de dor e trauma mamilar no grupo intervenção (aconselhamento), no entanto, aos dois e quatro meses, cerca de 93% das mulheres do grupo intervenção estavam seguras, a maioria sem problemas mamários e com produção suficiente de leite materno à demanda do RN, enquanto que mulheres do grupo controle representavam 32% com as mesmas características^(25)^. Dessa forma, o aconselhamento pode influenciar a puérpera no seu empoderamento, na sua resiliência e consequentemente, reduzir a crença de que seu leite é insuficiente, ou se detectada real insuficiência, superar esta dificuldade.

A revisão apontou que, embora o aconselhamento seja aplicável a todos os binômios, é particularmente útil em casos específicos, sendo enfatizada sua utilização para puérperas de gestação gemelares, com malformações mamárias/mamilares e/ou com dificuldades fisiológicas ou emocionais no processo de amamentação. A literatura também ressalta a necessidade de suporte através da abordagem do aconselhamento, especialmente para tabagistas^(28,31)^, obesas^(28,31)^, mulheres que voltarão a trabalhar^(28)^, nulíparas^(29,31)^, submetidas à cesariana^(29)^, gestações gemelares^(29)^, com problemas de saúde e/ou com necessidade de uso de medicamentos^(31)^, em casos de alterações na mecânica do aleitamento pelo neonato (prematuridade, fissuras, anquiloglossia) e em casos de lactogênese tardia^(31)^ e produção insuficiente^(31)^.

Ao analisar um estudo, observou-se que o termo “aconselhamento em AM” vem sendo utilizado, em alguns contextos, como sinônimo de educação em saúde, orientações e manejo clínico^(32)^. Tal fato pode ocasionar equívocos no mapeamento, interpretação e consolidação das evidências sobre a temática e dificuldade de incorporar as habilidades do aconselhamento nos protocolos institucionais e na prática clínica.

Estudo qualitativo com enfermeiros que atuam na promoção do AM apontou que os mesmos conhecem as estratégias para o manejo clínico da amamentação e que realizam a abordagem de forma humanizada. No entanto, suas ações não são sistematizadas, e muitas vezes o foco se restringe a orientações, com priorização apenas de binômios em alto risco^(33)^. Assim, o estudo aponta para a necessidade de capacitação profissional para o aconselhamento em aleitamento, uma vez que identificou-se que o apoio profissional eficaz caracteriza-se pelo fornecimento de cuidado desde o pré-natal por pessoal qualificado e capacitado, preferencialmente de modo presencial, com uso das habilidades de aconselhamento, com extensão para a rede de apoio, e com número de contatos entre quatro e oito^(10)^.

### Limitações do estudo

A escassez de estudos e de publicações recentes sobre a temática, assim como o maior detalhamento dos protocolos utilizados no aconselhamento, comprometem a comparabilidade dos resultados encontrados, o que consiste em uma limitação do estudo, ao mesmo tempo que aponta para a necessidade e potencialidade de novos estudos sobre a temática.

### Contribuições para as áreas da enfermagem, saúde ou políticas públicas

A revisão permitiu mapear e explorar os estudos sobre aconselhamento e sua aplicação durante a internação do binômio no AC, que pode nortear sua aplicação na prática, devido aos benefícios da estratégia, tal como servir de ponto de partida para novas pesquisas.

## CONCLUSÕES

Observou-se escassez de estudos que exploram a aplicação do aconselhamento em AM no AC. As evidências apontaram efeitos positivos do aconselhamento nos indicadores de AM, e a estratégia se mostrou uma ferramenta potente para o empoderamento materno e manejo da crença de leite insuficiente, assim como sua relevância nas singularidades. No entanto, o conceito de aconselhamento muitas vezes foi empregado nos estudos como sinônimo de orientações/educação em saúde, o que pode reduzir a proposta efetiva do conceito de aconselhamento. Há que se destacar ainda a escassez de descrição de protocolos de aconselhamento e das intervenções consideradas padrão nas instituições hospitalares e a necessidade de novos estudos sobre a temática.
